# Supplementary material for: Content-rich biological network constructed by mining PubMed abstracts
Source: BMC Bioinformatics. 2004 Oct 8;5:147. doi: 10.1186/1471-2105-5-147 (PMC528731; doi:10.1186/1471-2105-5-147)
Supplement: Additional File 5 — The original Chilibot query results of the term "long-term potentiation (LTP)" and 22 other terms, limiting the latest references analyzed to the years 1990, 1995, 2000, and 2004. [file 1471-2105-5-147-S5.bz2 › chilibotAdditionalFile5/ltp2000/html/PLC_SYNAPTOTAGMIN.html]

 


 **PLC** and **SYNAPTOTAGMIN** 
  
Found 2 abstracts in PubMed,  **2 abstracts were retrieved and analyzed**.  


---

 Search Google  |
 PDF files only 
|  EDU domain only 

---

**Interactive relationship** (e.g. stimulation, inhibition, etc)

- The similarity between the structures of the  **synaptotagmin**  I C2A domain and the  **PLC**  delta1 C2 domain suggests that the latter binds four calcium ions and acts by a similar mechanism.  Ref: 9819203 Biochemistry, 1998
- The C2 domain of  **PLC**  delta1 is a circularly permuted topological variant P variant of the  **synaptotagmin**  I C2A domain S variant.  Ref: 9062102 Biochemistry, 1997
